# Mycotoxins: Biotransformation and Bioavailability Assessment Using Caco-2 Cell Monolayer

**DOI:** 10.3390/toxins12100628

**Published:** 2020-09-30

**Authors:** Van Nguyen Tran, Jitka Viktorová, Tomáš Ruml

**Affiliations:** Department of Biochemistry and Microbiology, University of Chemistry and Technology, Technicka 3, 166 28 Prague 6, Czech Republic; tranv@vscht.cz (V.N.T.); prokesoj@vscht.cz (J.V.)

**Keywords:** permeability, bioavailability, intestinal transport, metabolism, mycotoxins, biotransformation, cytochrome

## Abstract

The determination of mycotoxins content in food is not sufficient for the prediction of their potential in vivo cytotoxicity because it does not reflect their bioavailability and mutual interactions within complex matrices, which may significantly alter the toxic effects. Moreover, many mycotoxins undergo biotransformation and metabolization during the intestinal absorption process. Biotransformation is predominantly the conversion of mycotoxins meditated by cytochrome P450 and other enzymes. This should transform the toxins to nontoxic metabolites but it may possibly result in unexpectedly high toxicity. Therefore, the verification of biotransformation and bioavailability provides valuable information to correctly interpret occurrence data and biomonitoring results. Among all of the methods available, the in vitro models using monolayer formed by epithelial cells from the human colon (Caco-2 cell) have been extensively used for evaluating the permeability, bioavailability, intestinal transport, and metabolism of toxic and biologically active compounds. Here, the strengths and limitations of both in vivo and in vitro techniques used to determine bioavailability are reviewed, along with current detailed data about biotransformation of mycotoxins. Furthermore, the molecular mechanism of mycotoxin effects is also discussed regarding the disorder of intestinal barrier integrity induced by mycotoxins.

## 1. Introduction

Mycotoxins are toxic secondary metabolites secreted by fungi and frequently occurring in food and feed worldwide [1,2,3]. The major fungal genera producing foodborne mycotoxins are *Fusarium*, *Aspergillus, Penicillium*, and *Alternaria* [4,5]. *Fusarium* is one of the most important producers of toxins falling into the three major classes of mycotoxins, such as fumonisins (FBs), zearalenone (ZEA), trichothecenes (deoxynivalenol (DON), nivalenol (NIV), and T-2 and HT-2 toxins, and also emerging mycotoxins involving beauvericin (BEA) and enniatins (ENNs) [6,7]. Ochratoxin A (OTA), the major mycotoxin of the ochratoxins, is produced by various species of the *Aspergillus* and *Penicillium* genus [8]. In addition, *Penicillium* species are known to produce mycophenolic acid (MPA) [9] and patulin (PAT) [10,11]. Aflatoxins, including aflatoxin B1 (AFB1), aflatoxin B2 (AFB2), aflatoxin M1 (AFM1), aflatoxin M2 (AFM2), aflatoxin G1 (AFG1), and aflatoxin G2 (AFG2), are the most studied group of mycotoxins produced by *Aspergillus flavus* [12,13]. *Alternaria* fungi contaminate a wide variety of food items, such as cereals, fruits, wheat, barley, and sorghum, producing several toxins, with alternariol (AOH), alternariol monomethyl ether (AME), tentoxin (TEN), tenuazonic acid (TeA), altenuene (ALT), and altertoxins (ATXs) being the most relevant ones [14,15]. The effects of mycotoxins on cell functions are listed in Table 1.

The naturally ubiquitous occurrence of mycotoxins in food has been widely documented (Table 2). Thus, mycotoxins can contaminate a variety of foodstuffs, such as grain-based products (wheat, oats, barley, maize, and rye), nuts, dried fruits, spices, cocoa, coffee, beer, wine, fruits, meat, and animal products (eggs, milk, and cheese) [14,46,47,48,49,50]. Some mycotoxins are thermostable, allowing them to endure most food processes [51]. DON is stable up to 120–180 °C and was degraded after 40 min at 210 °C [52]. For ZEA, heat treatment at temperatures up to 160 °C had no significant effect and 85% reduction of the toxin concentration in barley flours was achieved after 60 min at 220 °C [53]. DON content in whole and white breads decreased by 49% and 39%, respectively, compared to the original flours [51]. According to Generotti et al. [54], increasing pH and baking time in an acceptable technological range can reduce DON concentration in the final product [54]. T-2 and HT-2 are relatively stable during the thermal process up to 170 °C and about 45% of T-2 and 20% HT-2 were thermally degraded during biscuit-making [55]. Similarly, the loss of OTA, ZEA, AFB1, BEA, and ENNs in the products showed that thermal processing effectively decreased the content of these mycotoxins [52,56,57]. The 54% AOH reduction was reported for treatment at 110 °C for 20 min, whereas no significant effect was found for AME at the same temperature [58]. Based on Stadler et al. [59], the parent mycotoxin can be structurally modified during food processing, including isomerization, decarboxylation, rearrangements, and the reaction with other small molecules [59]. In summary, food processing such as brewing, cooking, baking, frying, canning flaking, nixtamalization, and extrusion, in general, reduced concentrations of some mycotoxins but did not eliminate them completely [52,59,60].

Ingestion of contaminated food is considered as a major route for exposure to many mycotoxins [51]. Upon ingestion, mycotoxins may induce local toxicity or cross the intestinal barrier to enter the bloodstream and reach target organs [2]. Nevertheless, to achieve any effect in a specific tissue or organ, the mycotoxins must be available in effective concentration at certain location, which refers to the compound’s tendency to be extracted from the food matrix, and they must then be absorbed from the gut via the intestinal cells [67]. The term bioaccessibility refers to the fraction of a mycotoxin liberated from a food matrix that passes unmodified through complex biochemical reactions related to the gastrointestinal digestion and thus becomes available for absorption in the small intestine [68,69]. Bioaccessibility can be considered as an indicator for the maximal absorption of the toxin, which can be used for realistic worst-case risk assessment of the toxin in a consumer product [70]. In fact, foodborne mycotoxins can be degraded or modified by metabolic processes of the human body, and only a fraction of the initial content can pass the intestinal membrane to enter the bloodstream [71]. In this sense, bioavailability is defined as the portion of ingested contaminant in food that reaches the systemic circulation [72].

To determine the bioavailability of mycotoxins, different in vitro models or in vivo experiments have been carried out. In vivo experiments would be the best way to evaluate the efficacy of binding capacities [73]. However, to avoid the ethically questionable use of animals in the in vivo experiments, the in vitro models have been used instead. The bioavailability studies carried out in animals are complex, expensive, and lengthy, while the in vitro experiments can be simple, rapid, and cost-effective [72]. The advantages and disadvantages of each procedure are summarized in Table 3. Most of the in vitro studies of the gut were done with human colon tumorigenic cell lines Caco-2, T84, TC7, and HT-29 [74]. The brief description of the expression of transporters, enzymes, and other relevant proteins of available cell lines used for the in vitro biotransformation and bioavailability of drugs and xenobiotics is stated in Table 4. Among commercially available cell lines, Caco-2 cells have been widely used to study absorption, metabolism, and bioavailability of drugs and xenobiotics [2,74]. This model is generally suitable for screening drug and nutrient compounds due to a good in vitro–in vivo correlation [75].

P-glycoprotein (P-gp), multidrug resistance protein (MRP), breast cancer resistance protein (BCRP), uridinediphosphoglucuronosyl transferase (UGT), sulfotransferase (SULT), *N*-acetyltransferase (NAT), glutathione–S–transferase (GST), and cytochrome P (CYP).

This review mainly focuses on the biotransformation of mycotoxins via the expression regulation of some critical enzymes and the currently available data regarding the in vitro study of the bioavailability of mycotoxins using the Caco-2 monolayer. Furthermore, the usefulness and limitations of this model are also discussed.

## 2. Biotransformation of Mycotoxins

**Figure 1:**
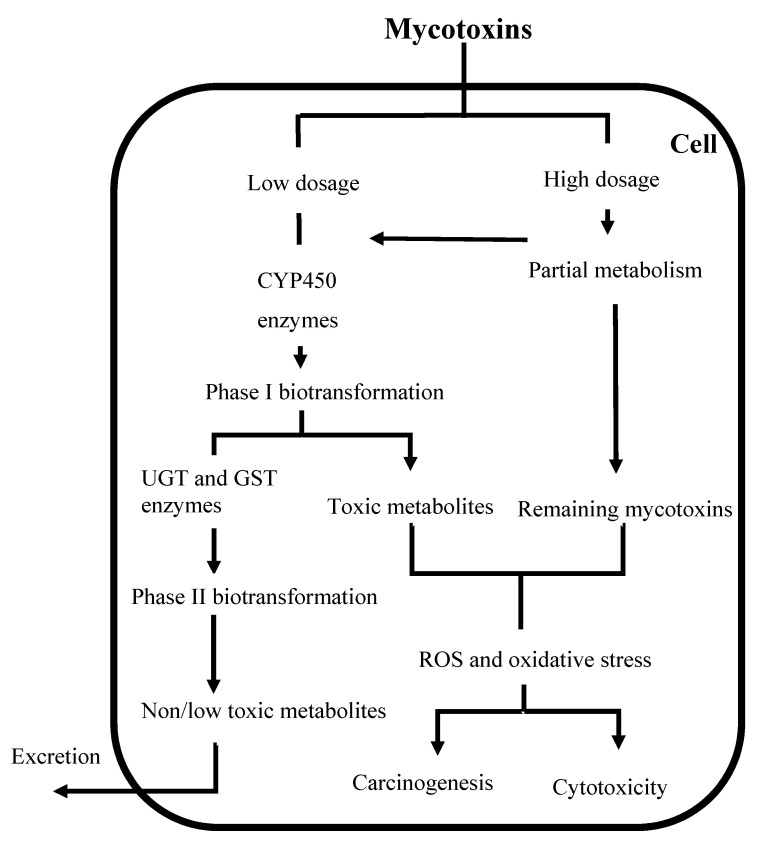
Major biotransformation and adverse cellular effects of mycotoxins. CYP450: Cytochrome P450; UGT: Uridine 5′-diphospho-glucuronosyltransferase; GST: Glutathione S-transferase; ROS: Reactive oxygen species.

Mycotoxins biotransformation is defined as all the complex modifications which alter the structure of mycotoxins by chemical reactions within the body [88]. Biotransformation is often referred to detoxification, but biotransformation enzymes can also convert certain chemicals into highly toxic metabolites (Figure 1) in a process known as bioactivation [89]. Biotransformation of mycotoxins involves two distinct stages, namely phase I and phase II. The biotransformation process allows metabolites created during phase I to enter conjugation processes (phase II), but in some cases, the substances may be eliminated directly after phase I [90].

In phase I, the mycotoxin could be oxidized, reduced, or hydrolyzed based on their chemical structure [90]. The enzymes involved in detoxification belong to the cytochrome P (CYP) superfamily. The CYP superfamily contains the enzymes involved in oxidative metabolism, such as monooxygenases, prostaglandin synthases, amine oxidases and alcohol dehydrogenases; and reductive metabolism mainly governed by epoxide hydrolases, and aldehyde or ketone reductases [91]. CYP450 enzymes play an important role in the oxidative and reductive metabolism of many endogenous or exogenous chemical compounds [34], including most mycotoxins (Table 5). In mammals, CYPs are present in the endoplasmic reticulum and mitochondria of most cells [89]. Among CYPs, CYP3A with an average content from 50–70% of total enteric CYPs is the major subfamily expressed in the human small intestine [92].

Phase II reactions are known as conjugation reactions, which usually refer to covalent binding of endogenous hydrophilic substances such as glucuronic acid and sulfate. The reactions provide more hydrophilic compounds, which are quickly eliminated. In general, phase II reactions decrease the toxicity [89]. Uridine 5′-diphospho-glucuronosyltransferase (UDP-glucuronosyltransferase–UGT) and glutathione S-transferase (GST) enzymes play an important role in the phase II metabolism [89,91].

Although the liver is the main detoxification organ, extrahepatic tissues in the gastrointestinal tract (GI tract), kidney, and bladder also show metabolic activity. The GI tract is a first physical barrier for mycotoxins but it also influences the biotransformation process and bioavailability of mycotoxins in other ways. Microorganisms from guts have been reported to exhibit the capacity for degrading mycotoxins [131,132,133,134]. Additionally, P-glycoprotein (P-gp) and multidrug resistance protein (MRP), members of the ATP–binding cassette (ABC) superfamily of transport proteins, are able to pump mycotoxins out of the intestinal cells, leading to limit bioavailability of the substrates [71,135]. Both CYP450 and P-gp in the gut play a crucial role in defense mechanisms against mycotoxins that reach the intestinal mucosa [92].

Previous biotransformation studies mainly focused on AFB1, OTA, trichothecenes (T-2 and DON), ZEA, and FBs. Recently, emerging *Fusarium* and *Alternaria* mycotoxins have gained more interest [46], although in vivo metabolization data are still limited. The biotransformation products of mycotoxins are summarized in Table 3. These studies revealed that mycotoxins can induce the expression of CYP450 enzymes in animal and human cell lines.

### 2.1. Biotransformation of Aflatoxins

**Figure 2:**
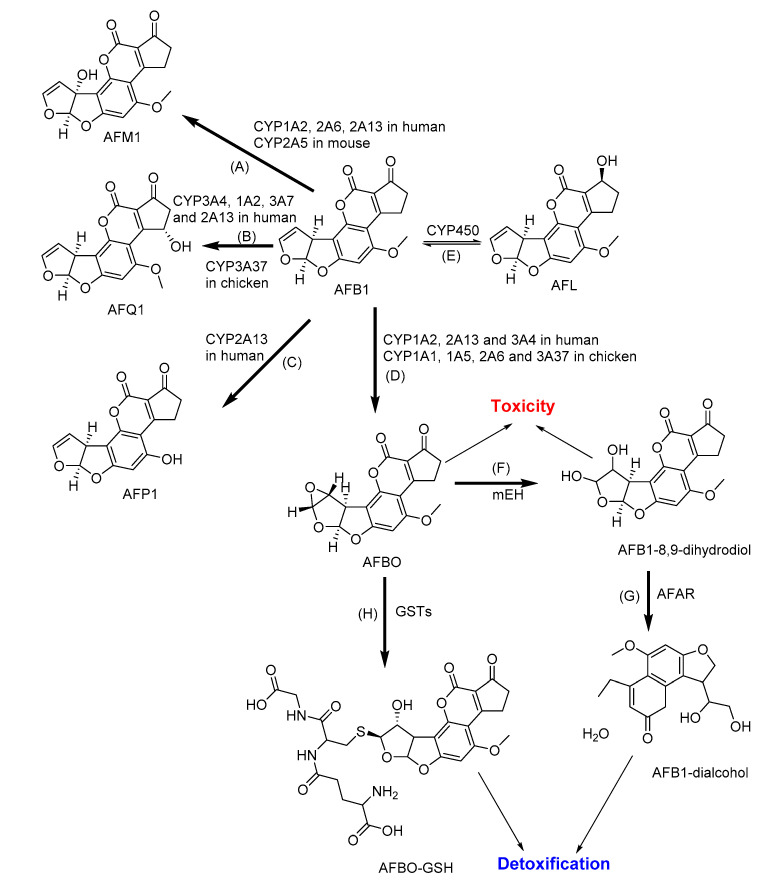
The major metabolic pathways of aflatoxin B1 (AFB1): (**A**) Aflatoxin M1 (AFM1) and (**B**) aflatoxin Q1 (AFQ1) by hydroxylation; (**C**) Aflatoxin P1 (AFP1) by demethylation; (**D**) AFB1–8,9-epoxide (AFBO) by epoxidation; (**E**) Aflatoxicol (AFL) by ketoreduction; (**F**) AFB1-8,9-dihydrodiol by microsomal epoxide hydrolase (mEH); (**G**) AFB1-dialcohol by aflatoxin-aldehyde reductase (AFAR); and (**H**) AFBO-glutathione (AFBO-GSH) by conjugation with glutathione. CYP: Cytochrome P; GSTs: Glutathione S-transferases [123].

Native AFB1 itself is not toxic, but the bioactivation by cytochrome CYP450 leads to AFB1-8,9-epoxide (AFBO), which is acutely toxic, mutagenic, and carcinogenic (Figure 2) [34]. Additionally, the metabolic pathway of AFB1 can also give rise to moderately toxic aflatoxicol (AFL) by ketoreduction, mildly toxic AFM1 and relatively nontoxic aflatoxin Q1 (AFQ1) by hydroxylation, and relatively nontoxic aflatoxin P1 (AFP1) by demethylation [123,136]. Their formation is thus considered as a detoxification pathway [137]. CYP enzymes, particularly CYP1A2 and CYP3A4, are predominant in the metabolic activation of AFB1 [122]. The detoxification of AFBO and AFM1 is realized by conjugation with glutathione catalyzed by GST. Otherwise, the unconjugated AFBO is alternatively hydrolyzed to AFB1-dihydrodiol, which is reversibly converted to AFB1-dialdehyde [34,90,138]. AFB1-dialdehyde is metabolized by the enzymes of aldo-keto reductase subfamily 7 (AKR7) and microsomal epoxide hydrolase (mEH) to form the nontoxic AFB1-dialcohol metabolite in humans, rats, mice, and pigs [123,139,140].

### 2.2. Biotransformation of Ochratoxin A

**Figure 3:**
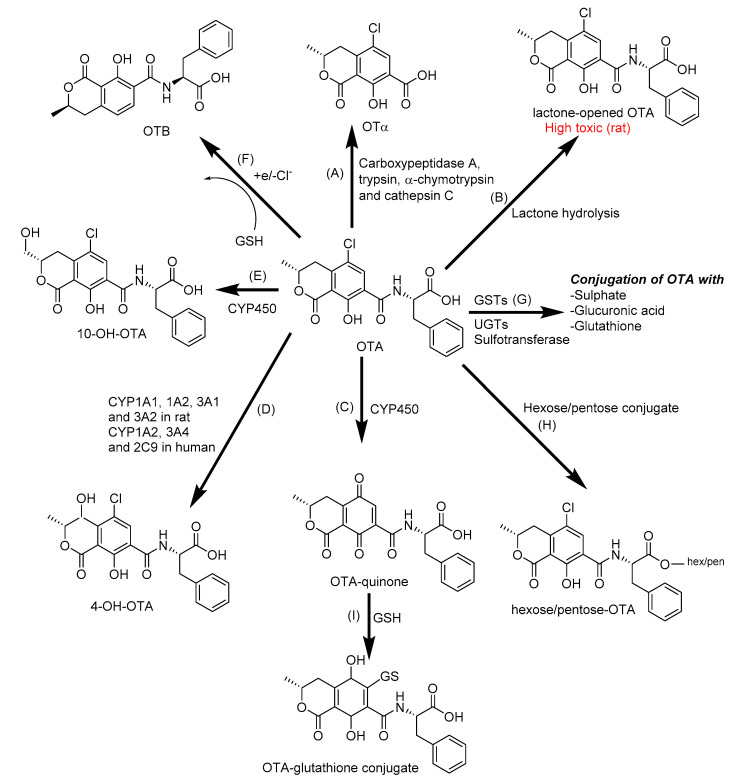
The biotransformation of ochratoxin A (OTA): (**A**) OTα by cleavage of the peptide bond of OTA; (**B**) lactone-opened OTA by lactone hydrolysis; (**C**) OTA-quinone by oxidation; (**D**) 4-hydroxy-ochratoxin A (4-OH-OTA) and (**E**) 10-hydroxyochratoxin A (10-OH-OTA) by hydroxylation; (**F**) OTB by dechlorination; (**G**) OTA-glutathione, OTA-glucuronide and OTA-sulfate by conjugation with glutathione (GSH), glucuronic acid, and sulfate; (**H**) Hexose/pentose-OTA by conjugation with hexose/pentose, (**I**) OTA-glutathione by conjugation with glutathione. CYP450: Cytochrome P450; GSTs: Glutathione S-transferases; UGTs: Uridine 5′-diphospho-glucuronosyltransferases [129].

In animals and humans, OTA can be metabolized by both phase I and phase II enzymes to many different products in the liver, kidney, and intestine (Figure 3). Poor biotransformation and slow elimination of metabolites contribute to the toxicity, carcinogenicity, and organ specificity of OTA [139,141]. In the gut, ochratoxin α (OTα), a major metabolite and is formed by carboxypeptidases, which cleave the peptide bond in OTA [34]. Other types of major metabolites of OTA are 4-hydroxy-ochratoxin A (4-OH-OTA) and 10-hydroxyochratoxin A (10-OH-OTA) have been identified from the urine of rats and are also produced by human, pigs, goat, chicken, rat, and rabbit liver microsomes or human bronchial epithelial cells in vitro [142,143,144]. Most of the metabolites of OTA, such as OTα, OTB, 4-OH-OTA, and 10-OH-OTA, are less toxic than the original compound [129,139]. However, opening the lactone ring under alkaline conditions (called the lactone-opened OTA), found in rodents, leads to more toxic metabolites than OTA itself [126]. These phase I-type reactions probably relate to the action of the CYP450 enzyme family, including CYP1A1, CYP1A2, CYP3A1, CYP3A2, CYP3A4, CYP3A5, CYP2B6, and CYP2C9 [124,125,126]. Phase II biotransformation mainly occurs in the liver with conjugation of OTA with sulfate, glucuronide, hexose/pentose, and glutathione [127,128,129].

### 2.3. Biotransformation of Deoxynivalenol

**Figure 4:**
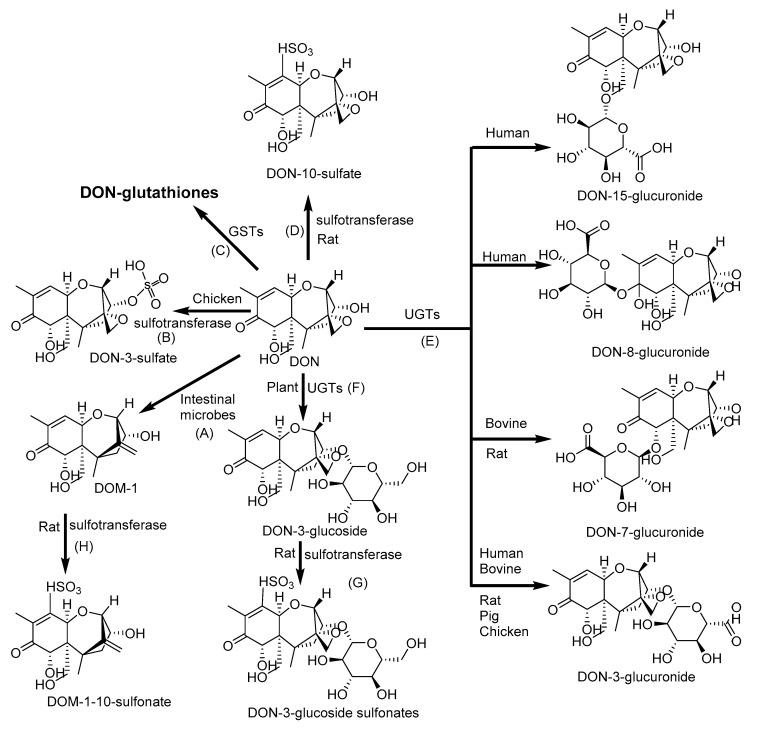
Phase II biotransformation of deoxynivalenol (DON): (**A**) Deepoxy-deoxynivalenol (DOM-1) by deepoxidation; (**B**) DON-3-sulfate, (**D)** DON-10-sulfate, (**G**) DON-3-glucoside sulfonate and (**H**) DOM-1-10-sulfonate by sulfation; (**C**) DON-glutathiones by conjugation with glutathione; (**E**) DON-3-glucuronide, DON-7-glucuronide, DON-8-glucuronide, and DON-15-glucuronide by conjugation with glucuronic acid; and (**F**) DON-3-glucoside by conjugation with glucose. GSTs: Glutathione S-transferases; UGTs: Uridine 5′-diphospho-glucuronosyltransferases [102].

DON is not substrate of phase I metabolism [145]. Major metabolites of DON include the glucuronide and sulphate conjugates of DON (Figure 4) and deepoxy-deoxynivalenol (DOM-1) [146]. DOM-1 showed lower cytotoxicity in pigs [147]. DON conjugates with glycosides or sulfonates to form DON-3-gluccoside (D3G); DON-, DOM- and D3G-sulfonates; and DON-3-, DON-7-, DON-8-, and DON-15- glucuronides identified in porcine, rat, chicken, bovine, and human [34,102,148,149,150]. Other DON-biotransformation products, including DON-glutathione conjugates and the products of glutathione degradation, such as DON-S-cysteinyl-glycine and DON-S-cysteine, have been reported in cereals. Thanks to intestinal microflora, DON could be metabolized in animals and humans but not deposited in the tissues [151,152].

### 2.4. Biotransformation of T-2 and HT-2

**Figure 5:**
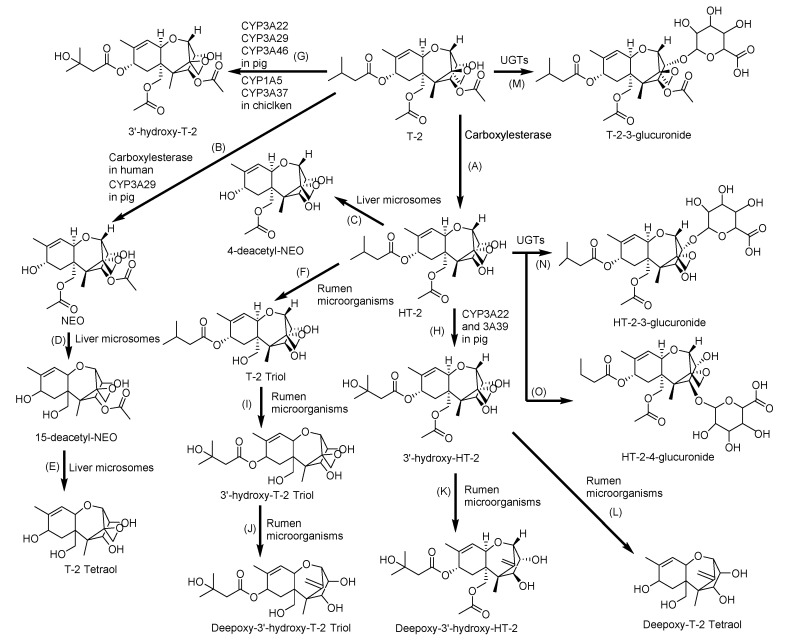
Metabolic pathway of T-2 toxin (T-2): (**A**) HT-2 toxin (HT-2), (**B**) Neosolaniol (NEO), (**C**) 4-deacetyl-NEO, (**D**) 15-deacetyl-NEO, (**E**) T-2 triol and (**F**) T-2 tetraol by hydrolysis; (**G**) 3′-hydroxy-T-2, (**H**) 3′-hydroxy-HT-2 and (**I**) 3′-hydroxy-T-2 triol by hydroxylation; (**J**) Deepoxy 3′-hydroxy-T-2 triol, (**K**) Deepoxy-3′-hydroxy-HT-2; (**L**) Deeopoxy-T-2 Tetraol by deepoxiadtion; and (**M**) T-2-3-glucuronide, (**N**) HT-2-3-glucuronide, and (**O**) HT-2-4-glucuronide by conjugation with glucuronic acid. UGTs: Uridine 5′-diphospho-glucuronosyltransferases [154].

The major metabolic pathways of T-2 include hydroxylation, hydrolysis, deepoxidation, and conjugation (Figure 5) [153]. The typical metabolites of T-2 in human and animals are HT-2 toxin (HT-2), neosolaniol (NEO), 3′-OH-T-2, 3′-OH-HT-2, T-2 triol, T-2 tetraol, and some C12,13-deepoxy products [99,154]. The contributions of the CYP450 enzymes to T-2 metabolism follow the descending order of CYP3A4, CYP2E1, CYP1A2, CYP2C9, and CYP2B6 or CYP2D6 or CYP2C19, in which CYP3A4 contributes the most [93]. In addition, CYP1A1 in human [34]; CYP3A46, CYP3A29, and CYP3A22 in pig [94,95,96]; and CYP1A5 and CYP3A37 in chicken [97,98] mainly convert T-2 to 3′-OH-T-2 and HT-2 to 3′-OH-HT-2 [145]. The carboxylesterase is also an important phase I enzyme, contributing to the rapid metabolism of T-2 to HT-2 [100]. A recent study revealed that cholic acid supplementation promotes the T-2 metabolism through activation of the farnesoid X receptor, which was found to have significantly increased the expression of CYP3A37 [99]. In phase II, glucuronidation of T-2 toxin, HT-2 toxin, and further phase I metabolites essentially contribute to the metabolism and excretion. The transformation of T-2 to T-2-3-glucuronide and HT-2 to HT-2-3-glucuronide and HT-2-4-glucuronide occurs in liver microsomes of rats, mice, pigs and humans [155]. The activities of GSTs and sulfotransferases can be also attributed to the conjugation reaction as a response to T-2 exposure [100,101].

### 2.5. Biotransformation of Fumonisins

**Figure 6:**
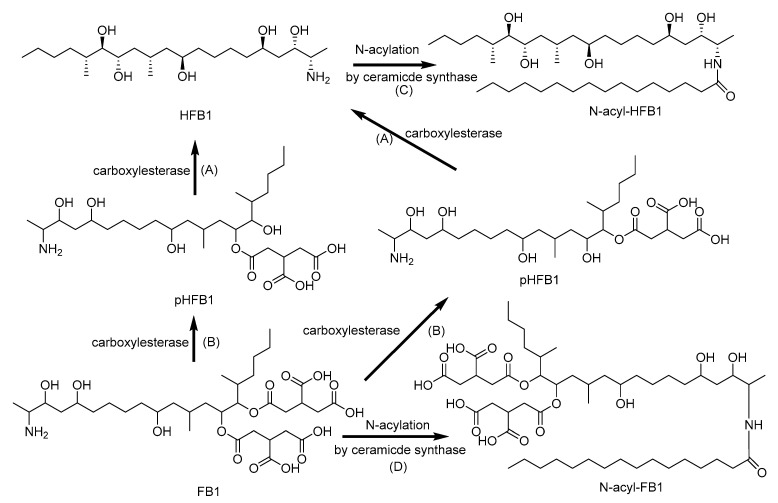
Metabolic pathway of fumonisin B1 (FB1): (**A**) Aminopentol (HFB1) and (**B**) partially hydrolyzed FB1 (pHFB1) by hydrolysis; (**C**) *N*-acyl-HFB1 and (**D**) *N*-acyl-FB1 by *N*-acylation [161,162,163].

After oral ingestion, FB1 are excreted primarily in the feces, either in the intact form or converted into aminopentol (HFB1) and partially hydrolyzed FB1 (pHFB1) by the intestinal microbiota (Figure 6) [116]. The supplementation with fumonisin carboxylesterase FumD results in the gastrointestinal degradation of FB1 and is considered as an effective strategy to detoxify FB1 in the digestive tract of turkeys and pigs [156]. The findings of Daud et al. [157] provided evidence that human fecal microbiota are capable of FB1 degradation, and LC-MS/MS fragmentation patterns indicated microbial biotransformation to hydrolyzed and partially hydrolyzed FB1 [157]. FB1 is not metabolized by CYPs. Moreover, it is a selective inhibitor of CYP2C11 and CYP1A2, while the activities of CYP2A1:2A2, CYP2B1:2B2, CYP3A1:3A2, and CYP4A are not significantly affected. The significant inhibition of CYP2C11 might be related to suppressed protein kinase activity as a result of the inhibition of sphingolipid biosynthesis caused by FB1 [158,159,160]. FB1, HFB1, and pHFB1 can be acetylated to form *N*-acetylated fumonisins with fatty acid of various lengths, and *N*-acyl forms proved to be more toxic than the parent FB1 [161,162,163].

### 2.6. Biotransformation of Zearalenone

**Figure 7:**
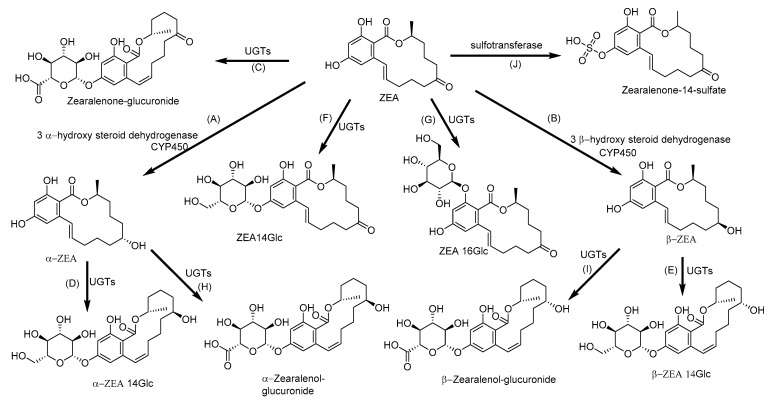
Metabolic pathway of zearalenone (ZEA): (**A**) α- zearalenol (**B**) (α-ZEA) and β- zearalenol (β-ZEA) by hydroxylation; (**C**) Zearalenone-glucuronide, (**D**) α-zearalenol-glucuronide and (**E**) β-zearalenol-glucuronide by glucuronidation; (**F**) Zearalenone-14-glucoside (ZEA14Glc), (**G**) Zearalenone-16-glucoside (ZEA16Glc), (**H**) α- zearalenol-14-glycoside and (**I**) β-zearalenol-14-glucoside by glycosidation; and (**J**) Zearalenone-14-sulfate by sulfation. UGTs: Uridine 5′-diphospho-glucuronosyltransferases [170].

ZEA is mainly biotransformed into α-zearalenol (α-ZEA), which shows the highest binding affinity to human and porcine estrogen receptors, whereas in broilers and rats, β-zearalenol (β-ZEA) with the low affinity to the receptor is predominantly produced [103,104,105]. ZEA upregulates mainly mRNA levels of CYP2B6, CYP3A4, CYP1A2 and CYP1A1, followed by CYP3A5 and CYP2C9, together with activation of their transcriptional regulators—aryl the hydrocarbon receptor (AhR), constitutive androstane receptor (CAR), and pregnane X receptor (PXR) [106]. It is well known that ZEA, α-ZEA, and β-ZEA are substrates of UGT, the enzyme responsible for the glucuronidation (Figure 7) [78,105,164,165]. However, the UGT was not only saturated but also inhibited by high concentration of ZEA [166]. Although zearalenone-14-glucoside (ZEA14Glc) has lower toxicity than ZEA due to inability to interact with estrogen receptors, the possible systemic hydrolysis and further activating metabolism of ZEA14Glc leads to ZEA-mediated toxicity [167]. Due to the adverse effect of ZEA on human and animal health, microorganisms have gained great interest in the modulation of ZEA adsorption and transformation [168,169]. Eukaryotic cells were able to biotransform ZEA to α-ZEA and β-ZEA, while prokaryotic cells only absorbed ZEA without any metabolization of this mycotoxin and sequestered ZEA by binding to the cell wall [170,171].

### 2.7. Biotransformation of Enniatins

**Figure 8:**
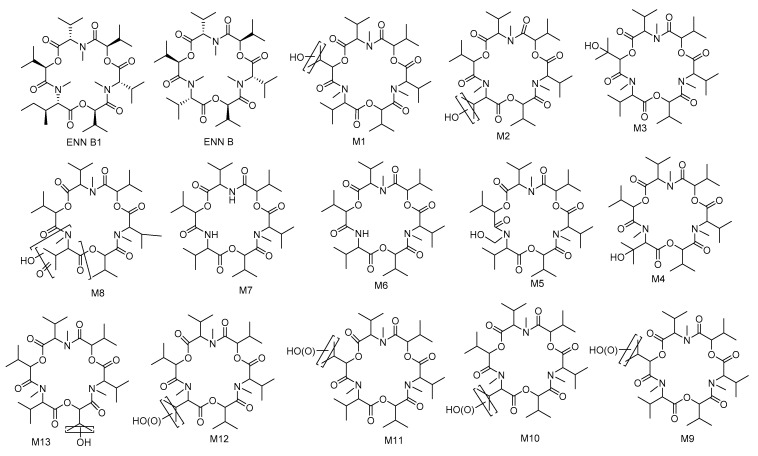
Molecular structures of ENN B, and B1, and proposed structures of their metabolites [110,111].

**Figure 9:**
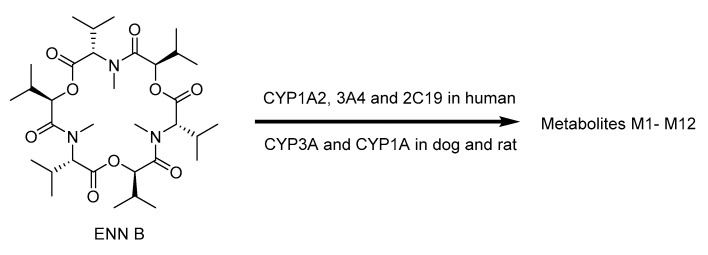
The biotransformation of ENN B [110,111].

For ENNs, the most information is currently available for ENN B and B1. In vitro and in vivo studies demonstrated that CYP3A4, CYP2C19, and CYP1A2 play the major role for ENN B metabolism in human microsomes and CYP3A and CYP1A are also included in this process in rats and dogs [109]. The 12 biotransformation products were characterized after the incubation of ENN B with rat, dog, and human liver microsomes (Figure 8): M1–M5 were monohydroxylated and M6 and M7 were *N*-demethylated, whereas M8–M12 were the result of multiple oxidations [110]. However, only eight metabolites could be detected in the case of chicken liver microsomes, particularly five hydroxylated (M1–M5) and three carboxylated (M9, M11 and M12) metabolites. Moreover, M4 and M13 were major metabolites in egg samples, while M11 and M13 were found in liver and serum samples collected after broilers and hens were given contaminated feed containing ENN B [111]. Similarly, ENN B1 is mainly metabolized by CYP3A4 [112]. In vitro incubation with minipig and slaughter swine liver microsomes resulted in the detection of ten ENN B1 metabolites (M2–M11) and M1 occurred only in the minipig assays, while six metabolites (M5–M8) were detected also in vivo [113]. Rumen microbiota also proved to be able to degrade ENN B up to 72% after 48 h of incubation [114]. Any sulfated or glucuronidated phase II metabolites of ENN B or ENN B1 were detected (Figure 9) [115].

### 2.8. Biotransformation of Beauvericin

**Figure 10:**
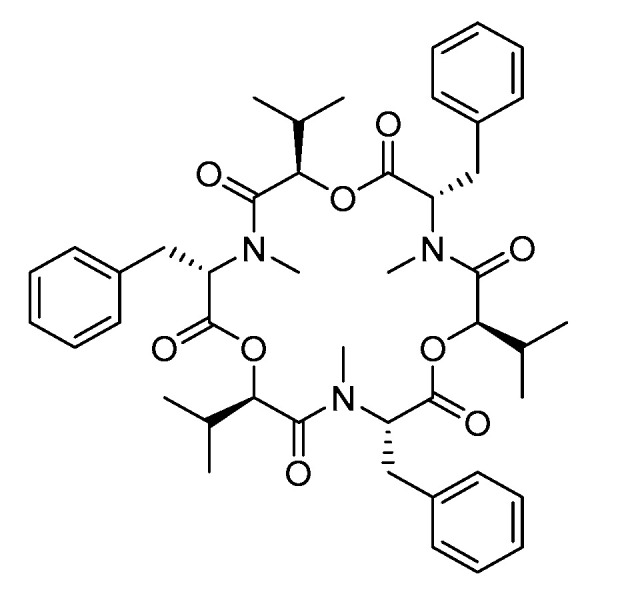
Molecular structure of BEA.

Very few studies have been carried out on BEA (Figure 10) in this regard. No BEA metabolites were detected in the mice feed with BEA in the study of Rodríguez-Carrasco et al. [107], suggesting a higher metabolic stability for BEA [107]. Mei et al. [108] reported that BEA is a potent inhibitor of diverse CYP450 enzymes, including CYP3A4/5 and CYP2C19 in human liver microsomes and CYP3A1/2 in rat liver microsomes [108].

### 2.9. Biotransformation of Alternaria Mycotoxins

**Figure 11:**
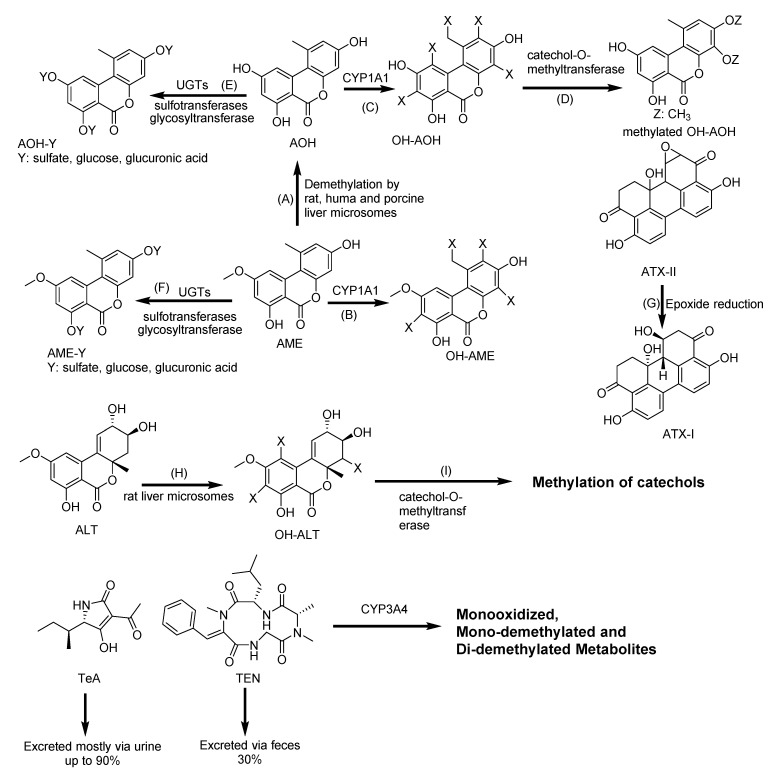
Biotransformation pathway of *Alternaria* mycotoxins: Alternariol (AOH), alternariol monomethyl ether (AME), hydroxy-alternariol (OH-AOH), hydroxy-alternariol monomethyl ether (OH-AME), tenuazonic acid (TeA), altertoxins (ATXs), Tentoxin (TEN), altenuene (ALT), hydroxyl-altenuene (OH-ALT). (**A**): Demethylation; (**B**,**C**,**H**): Hydroxylation; (**D**,**I**): Methylation; (**E**,**F**): Sulfation, glycosylation, and glucuronidation; (**G**): Epoxide reduction. CYP: Cytochrome P; and UGTs: Uridine 5′-diphospho-glucuronosyltransferase [71,117,121,172,173,174,177,178,179,180,181].

AOH and AME form the metabolites hydroxylated at C-2, C-4, and C-8 by activation of the CYP1A1 enzyme (Figure 11) [172,173]. AOH and AME activate the AhR pathway, which induces CYP1A1 expression [117,118]. AOH is known for its genotoxicity [118]. However, the phase I metabolites, 4-OH-AOH and 4-OH-AME, had minor effect compared to AOH or AME in topoisomerase inhibition and DNA strand-breaking effects [174]. Phase II metabolism includes conjugation with glucuronic acid and sulfate [119]. AME and AOH were enzymatically glycosylated using whole-cell biotransformation system, producing highly effective rates of 58% AOH-3-glucoside, 5% AOH-9-glucoside, and 24% AME-3-glucoside [120]. However, human gut microbiota was not capable of metabolizing AOH, AME, and ALT [175]. The conversion of ATX-II, significantly more genotoxic than AOH, to ATX-I by de-epoxidation in Caco-2 cells did not showed an adequate detoxification but an attenuation of genotoxicity [176]. The metabolic pathway of AOH, AME and other *Alternaria* mycotoxins, such as TEN, TeA, ALT and ATXs, are summarized in Figure 11.

### 2.10. Biotransformation of Patulin

**Figure 12:**
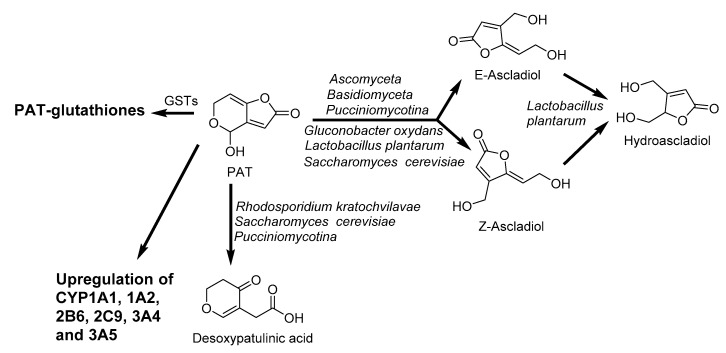
Biotransformation pathway of patulin (PAT): E-ascladiol, Z-ascladiol, hydroascladiol, and desoxypatulinic acid by microorganism, and PAT-glutathiones by reaction with glutathione. GSTs: Glutathione S-transferase [130,182,183,185,186].

PAT induces the upregulation of PXR and AhR accompanied by the enhancement of CYP1A1, CYP1A2, CYP2B6, CYP2C9, CYP 3A4, and CYP3A5 expression [130]. Moreover, PAT reacts with intracellular glutathione in gastrointestinal mucosa cells [182,183]. The extracellular enzymes of *Lactobacillus casei* YZU01 induced by PAT mainly degrades PAT, and the cell wall of this bacteria can also absorb a small amount of PAT [184]. Similarly, the degradation of PAT was observed by *Saccharomyces cerevisiae* during cider fermentation into E-ascladiol and Z-ascladiol (Figure 12), which are not toxic to human [185]. The biotransformation of PAT in humans and animals is not well understood and remains to be established.

## 3. Assessment of Bioavailability of Mycotoxins Using Caco-2 Cell Monolayer

The Caco-2 cell line is the most common and extensively used in vitro model to study the intestinal absorption of mycotoxins via the intestinal membrane enterocytes [2,10,187,188]. It was originally derived from a heterogeneous human epithelial colorectal adenocarcinoma cells established by Fogh and coworkers in 1977 [189]. The Caco-2 cells have the ability to spontaneously differentiate into a monolayer of cells, expressing many properties typical of absorptive enterocytes with a brush border layer, tight junctions, and efflux and uptake transporters as found in the small intestine [190,191,192]. Moreover, several phenolic compounds (e.g., kaempferol) are able to regulate the MAPK pathway, which is beneficial to the barrier functions [193]. Kaempferol treatment showed significant an increase in claudin 3, claudin 4, and occluden [194]. On the other hand, several mycotoxins—deoxynivalenol, zearalenone, fumonisin B1, T-2 toxin, aflatoxin M1, and ochratoxin A—have a deleterious effect on tight junctions of claudin 3, claudin 4, claudin 7, and occluden [195,196,197,198].

The Caco-2 cells have been shown to be a suitable model for biotransformation study because they express various phase-I hydroxylation and phase-II conjugation enzymes, and transport proteins of the ATP-Binding Cassette (ABC) superfamily [166]. Furthermore, a good correlation has been found for data on oral absorption in humans and the results in the Caco-2 model [199].

To closer mimic the intestinal barrier in vivo, Caco-2 cells were seeded on permeable membranes to form a confluent monolayer with a well-defined tight junction for approximately 21 days post-seeding [78]. The integrity of the Caco-2 monolayer was monitored by measuring the transepithelial electrical resistance (TEER), or by examining the permeability of paracellular markers, such as mannitol, inulin, Dextran, PEG 4000, Lucifer yellow, and phenol red [191,200]. Studies that have investigated the bioavailability of mycotoxins by Caco-2 cells are listed in Table 6. The results of these studies show that mycotoxins are transported through Caco-2 monolayer in different efficiencies.

DON, NIV, ZEA ENNs, and BEA cross easily the cell barrier. DON is efficiently transported through the intestinal barrier possibly either by passive/facilitated diffusion [202] or by paracellular passage through intercellular tight junctions [207]. All of the apparent permeability (P_app_) values greater than 1 × 10^−6^ cm/s suggest that these mycotoxins were absorbed efficiently [208]. P_app_ values for DON have been reported by many researchers. Sergent et al. [207] reported an average P_app_ value of 5.02 × 10^−6^ cm/s for absorption (apical (AP)–basolateral (BL) compartment) and excretion (BL–AP direction) [207]. In other study, absorption and excretion P_app_ values ranged 1.23–2.06 × 10^−6^ and 2.68–2.8 × 10^−6^ cm/s, respectively [202]. Finally, P_app_ value of 3.3 × 10^−6^ and 2.8 × 10^−6^ cm/s for absorption and excretion, respectively, were determined in study of Kodota et al. [209]. A faster bidirectional transport of DON in the mixture comparing to pure DON was observed, suggesting that the presence of other mycotoxins including AFB1, FB1, and OTA may promote intestinal transport of DON [210]. For NIV, transcellular transport probably occurred by passive diffusion in the absorptive direction, and P_app_ values were also higher than 10^−6^ cm/s [203]. The P_app_ values obtained with a concentration of 20 µM ZEA in the apical compartment and an incubation time of 1 h were 10.47 ± 4.7 × 10^−6^ cm/s [211]. About 30% of initial ZEA crossed the cell monolayer after 3 h of exposure, and 40% of ZEA was absorbed by the intestinal after 8 h [78]. ZEA presented higher bioavailability than its metabolites, α-ZEA, ranging from 10% to 36% (0–4 h; 30 µM) [72]. Unlike DON-3-glucoside (neither absorbed or cleaved by Caco-2 cells), ZEA-14Glc and ZEA-16Glc could cross the cell barrier and be absorbed by Caco-2 cells, resulting in further cleavage and the subsequent release of their parent deglycosylated forms [212]. BEA bioavailability was variable from 50% to 54% [213]. Higher duodenal bioavailability compared to colonic bioavailability of ENNs was observed. Particularly, the duodenal bioavailability of ENNs ranged from 58% to 77% for ENN A, from 69% to 70% for ENN A1, from 65% to 67% for ENN B, and from 62% to 65% for ENN B1. Colonic bioavailability ranged from 17% to 33% for ENN A, from 41% to 50% for ENN A1, from 48% to 55% for ENN B, and from 52% to 57% for ENN B1 [67]. In contrast, FB1 was not absorbed by Caco-2 cells [214].

Berger et al. [215] showed that OTA was absorbed by the human intestinal mucosa by passive diffusion of the undissociated form of OTA and it was not appreciably metabolized by Caco-2 cells [215]. DON and NIV were not significantly metabolized or accumulated in Caco-2 cells as well [71,202,203,207,216,217]. Therefore, upon ingestion, these mycotoxins can be absorbed from the gut via intestine cells, then entered into the systemic circulation and thus transported to the whole body. Nevertheless, the intestinal absorption of OTA would be limited thanks to the presence of the MRP2 [215] and breast cancer resistance protein (BCRP) [204]. An efflux of AFB1 was also associated with BCRP [218], and DON was a substrate for both P-gp and MRP2 [202]. P-gp has been shown to be involved in the efflux of FB1 [214], and NIV interacted with P-gp and MRP2 [203]. Several studies showed that DON transport was unaffected by the transporter [207,209]. However, DON uptake and efflux are carrier-mediated processes, and P-gp and organic anion-transporting peptides may be the major efflux/uptake transporters for DON in Caco-2 cells, respectively [219]. The stepwise c-Jun-*N*-terminal kinase–Akt–nuclear factor kappa-light-chain-enhancer of activated B cells (JNK-Akt-NF-κB) pathway elaborates upon P-gp induction following DON exposure in mammalian cells and provides a self-protection mechanism to resist exogenous toxic compounds such as DON and T-2 [220]. These dissimilarities may be consequences of differences in exposure conditions to the toxin. Particularly, transport experiments were performed in pH gradient, and the acidification of the apical compartment may increase the fraction of the uncharged molecules facilitating diffusion across the cell membrane and intracellular accumulation [221]. Furthermore, differences in the culture medium, passage number, and time in culture before splitting may lead to significant differences in ABC transporter expression and functionality [222].

Intestinal absorption of AOH was more extensive and faster than AME. About 23–26% of the apically applied AOH reached the basolateral compartment, while only about 3–7% of the initial amount of AME in the apical chamber reached the basolateral side. In basolateral medium, several metabolites were also detected: Three AOH metabolites (3-O-sulfate, 3-, and 9-O-glucuronide) and AME-3-O-glucuronide [119]. Several authors have already shown the ability of Caco-2 cells to metabolize ZEA into α- or β-ZEA, as well as into its glucuronidated and sulphated forms [78,166,211]. Videmann et al. [206] established that facilitated or active transport was involved in the transportation of ZEA and its metabolites. Particularly, they were substrates for ABCC1–3 transporters. ZEA and α-ZEA were mostly extruded by ABCC2 at the AP side and ABCC3 was able to transport β-ZEA at the BL side [206].

Treatment of Caco-2 cells with mycotoxins at reasonable concentrations must have no significant effect on cell viability, cell damage, and barrier integrity. FB1 at a concentration of up to 138 µM did not induce variation on cell viability and differentiation [214]. Similarly, ZEA concentration of up to 200 µM had no significant effect on cell viability and cell damage [78,206], and the integrity of the cell monolayers was preserved throughout the incubation with ZEA at a concentration of up to 40 µM, indicating that ZEA does not have detrimental effects on epithelial integrity in vitro [212]. Moreover, Caco-2 cells exposure to 5 µM of NIV showed neither a significant increase in the sucrose flux nor a significant decrease in TEER values [203]. DON also had no significant effect on Caco-2 cell viability at a concentration of up to 33 µM [202,209].

However, other studies reported that mycotoxins such as ZEA, DON, FB1, T-2, PAT, AFB1, and OTA decreased the TEER of intestinal epithelial cell lines in porcine as well as in human epithelium [10,195,196,198,223,224,225,226,227]. A reduction in TEER can cause an increase in the paracellular permeability, changes in transcellular flux through altered plasma channels or pumps, and uncontrolled cell death within the monolayer [228]. Pfeiffer et al. [211] showed that 20 μM of ZEN was able to affect the apparent permeability coefficients of Caco-2 cells, leading to their quick absorption from the intestinal lumen into the portal blood [211]. Moreover, the important indicators of intestinal permeability are tight junction proteins, which are comprised of several multiprotein complexes, including transmembrane proteins (claudin, occludin, and junctional adhesion molecule) and cytoplasmic scaffolding protein and signaling proteins, including zonula occludens [229]. DON, ZEA, FB1, T-2, AFM1, and OTA have a deleterious effect on tight junctions of claudin 3, claudin 4, claudin 7, and occluden [195,196,197,198].

**Figure 13:**
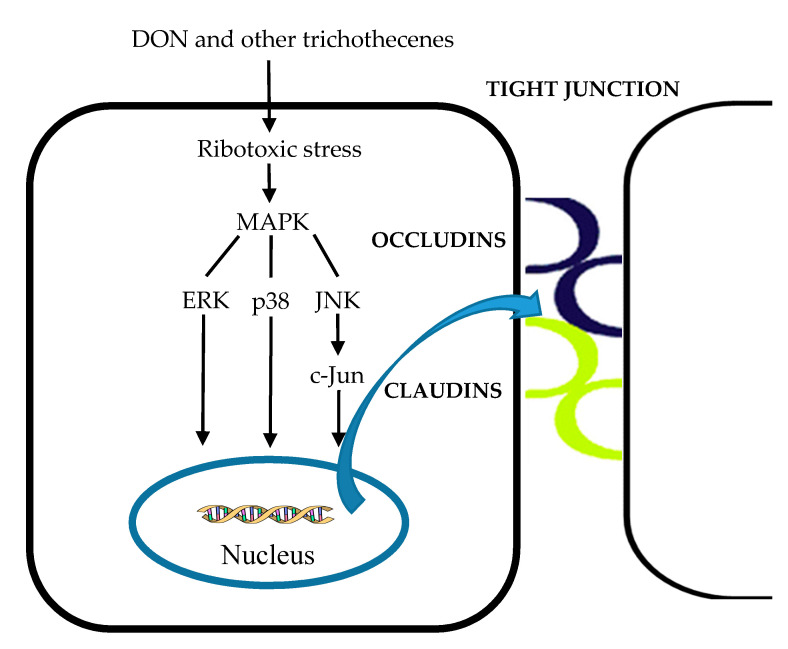
The effects of DON and other trichothecenes on the tight junction through activation of the MAPK pathway. MAPK: Mitogen-activated protein kinase-dependent, ERK: Extracellular signal regulated protein kinase, JNK: C-Jun-*N*-terminal kinase. The colored curves represent junction proteins.

Tight junction structure and function can be regulated by signaling molecules involved in the mitogen-activated protein kinase-dependent (MAPK) pathways [230]. Therefore, the rapid activation of MAPK, ZEA, and DON decreased the expression of tight junction proteins, resulting in intestinal barrier impairments [134,197]. DON and other trichothecenes are known for their binding of the ribosomal peptidyltransferase, inhibition of protein synthesis, and rapid activation of MAPK via inducing two signal transduction pathways of a process named the ribotoxic stress response [227,231,232,233]. The first pathway consists of the double-stranded RNA-activated protein kinase, leading to stimulation of JNK and p38 [25]. The second pathway involves hematopoietic cell kinase belonging to the Src tyrosine kinase family, which are upstream transducers of activation of MAPK. Among the primary MAPK subfamilies, such as p44/42 extracellular signal-regulated protein kinase (ERK), p38, and JNK [234], p44/42 ERK can be involved in intestinal epithelial cell morphology and in the structure of tight junctions. It was reported that the DON-induced activation of the p44/42 ERK signaling pathway inhibits the expression of claudin-4, which leads to reduces the barrier function of the intestine evaluated by TEER, paracellular permeability [197,227]. Treatment with 10 µM of DON also increased ERK, P38, JNK, and c-Jun phosphorylation levels by 2-fold, 30-fold, 61-fold, and 5-fold, respectively, and altered the gene expression levels of occludin, claudin-3, and the composition of tight junction proteins (Figure 13) [235]. The activation of p44/42 MAPK was partially involved in the detrimental effects of the integrity of tight junction caused by AFM1 and OTA [224].

In addition to the tight junction, the maintenance of intestinal barrier-related paracellular secretions, such as cytokines and chemokines, are important as well. ZEA metabolites, α- and β-ZEA, can be beneficial to the intestine by decreasing the expression of both interleukin-8 (IL-8) and interleukin-10 in a dose-dependent manner. Its metabolites have a rather anti-inflammatory effect on the epithelial intestinal cells [225]. However, cytokines are related to the impairment of intestinal integrity when exposed to ZEA and FB1 [225,226,236]. Moreover, the correlation between permeability and IL-8 secretion induced by DON in the intestine was investigated by the authors of [209]. IL-8 was examined as a factor affecting intestinal barrier function, and the increased IL-8 secretion may be involved in the TEER decrease [237]. Similar results were reported by the authors of [238]. Consequently, exposure to certain mycotoxins, particularly DON, may cause damage to the intestinal integrity and lead to various chronic intestinal inflammatory diseases, such as inflammatory bowel disease [195]. In addition, the synergic effects of OTA and AFM1 that might exacerbate intestinal inflammation were also reported [239].

Although the Caco-2 cells model offers several advantages, such as the reproducibility of results, controlled environment, and in-depth mechanistic insight [240], some limits of Caco-2 for assessing the bioavailability were also reported [241]. The main disadvantages of these models are the lack of the regulatory processes of the complex mucosal barrier and inability to accurately calculate the fractional transport and flux rate through the static transport conditions [242]. Moreover, it has been shown that significant variation of the expression level of efflux transporters, such as BCRP, MRP2, and MDR1 in the Caco-2 cell monolayer in human small and large intestines, affect the results as well [243,244]. The Caco-2 cell monolayer is somewhat unsuccessful in simulating in vivo intestinal environment due to lack of expression of CYP3A4, which is responsible for the biotransformation of many compounds in the human epithelial cell [245]. Further drawbacks of these models include the incapability of simulating the changes of intestinal pH system, since it is performed at constant pH conditions. In addition, variations in TEER and permeability were also reported to be related to the source of Caco-2 cell and interlaboratory differences in protocol design [192].

To reduce the heterogeneity of the Caco-2 parental cell line and to improve the performance and the stability of this cellular model, some clonal derivative of Caco-2 cells have been established. The Caco-2/TC7 cell line, which was isolated from a late passage of the parental Caco-2 line, is suitable for intestinal absorption model due to a less heterogenic cellular population, resulting in better reproducibility of results [246]. The human intestinal HT-29 cell line is another cell line from colorectal origin with epithelial morphology and has a large proportion of mature goblet cells that can produce mucins. Therefore, the co-culture of Caco-2 and HT-29 with a ratio of 9:1 was used to provide a better representation of the intestinal tract [247]. In addition, the human colon carcinoma (HCT-116) and human colon adenocarcinoma (SW480) cells used in unraveling cancer-related mechanisms and the human duodenum adenocarcinoma (HuTu-80) cell line simulating duodenal cells are less popular [191]. More recently, a combination of in vitro digestion and Caco-2 absorption was used to simulate the physiological settings in the gastrointestinal tract and determine the bioaccessibility and bioavailability of the ZEA reaction products [72].

## 4. Conclusions

Scientific insights in the production of mycotoxins, their toxicities, biotransformation, and metabolism in different organisms have greatly contributed to a more detailed understanding of the chemical hazards in food. Mycotoxins can notably biotransform and detoxify in the liver, as well as in the digestive tract. The results obtained with Caco-2 monolayer are useful in the prediction of mycotoxins’ intestinal permeability, transport mechanism, and gene regulation of transporters and enzymes in humans, and may help interpret properly data of mycotoxins’ absorption for better comprehension of their possible adverse effects. Furthermore, the combined usage of in vitro digestion models with in vitro intestinal absorption models using Caco-2 cells may offer more complete picture during digestion in the intestinal tract. However, the correlation between in vitro Caco-2 data and in vivo situation necessitates further investigation.

## Figures and Tables

**Table 1:** Toxic effects of mycotoxins.

Mycotoxins	Effects	LD_50_ (mg/kg)	References
T-2 and HT-2	Inhibition of DNA, RNA and protein synthesis. Induction of mutations and apoptosis.	T-2 Rodents: 5–10 Pig: 5 Chicken: 2–6 Shrimp: 30 Mice: 2–5 HT-2 Rodents: 5–10	[16,17,18,19,20,21]
DON	Inhibition of DNA, RNA and protein synthesis. Decrease of the cell proliferation.	Mice: 46–78 Duck: 27 Chicken: 140	[22,23,24,25,26]
ZEA	Activation of the estrogen receptor. Inhibition of DNA and protein synthesis. Triggering lipid peroxidation and cell death.	Mice: 2000–20,000 Rat: 4000–10,000 Pig: 5000	[27,28,29]
BEA	Increase of the biological membrane permeability. Loss of ionic homeostasis. Induction of lipid peroxidation.	Mice: 100	[30,31,32]
ENNs	Increase of the membrane permeability for cations.	No acute in vivo toxicity data	[32,33]
FB1	Inhibition the activity of ceramide synthase.	>1000	[34]
AOH and AME	Single and double strand DNA breaks. Decrease of the cell proliferation.	Mice: 400 for AOH and AME	[35,36,37]
ATXs	DNA strand breaks.	Mice: 0.2	[37,38]
TeA	Inhibition of protein synthesis. Inhibition of photosynthetic activity.	Mice: 81(female), 186–225 (male) Rat: 168 (female), 180 (male)	[39,40,41,42]
AFB1	Damage of DNA Inhibition of protein synthesis through interfering with RNA transcription and translation. Induction of oxidative stress.	Swine: 0.62 Duck: 0.37 Turkey: 0.5–1 Chicken: 6.5–12.5 Quail: 19.5	[22,43]
MPA	Inhibition of inosine 5′-monophosphate dehydrogenase. Blocking of the DNA synthesis and proliferation of both T and B lymphocytes.	Rat: 450 Mice: 1900	[40,44]
OTA	Inhibition the activity of many enzymes which use phenylalanine as a substrate. Disruption of phenylalanine metabolism. Production of reactive oxygen species Lipid peroxidation, cell membranes and DNA damage	Dog: 0.2 Pig: 1 Chicken: 3.3 Rat and mouse: 20–50	[34,45]

T-2 toxin (T-2), HT-2 toxin (HT-2), deoxynivalenol (DON), zearalenone (ZEA), beauvericin (BEA), enniatins (ENNs), fumonisin B1 (FB1), alternariol (AOH), alternariol monomethyl ether (AME), altertoxins (ATXs), tenuazonic acid (TeA), aflatoxin B1 (AFB1), mycophenolic acid (MPA), and ochratoxin A (OTA). LD_50_: Median lethal dose.

**Table 2:** Occurrence of mycotoxins in food commodities.

Mycotoxins	Commodity	Concentration Range (µg/kg)	Country	References
T-2 and HT-2	Barley grain	26–787	Italy	[61,62]
	Maize	146	Hungary	
	Cereal-based products	<LOD-209	Tunisia	
	Wheat	6.7–15.2	Spain	
DON	Cereal and corn	96–1790	Portugal	[63,64]
	Wheat-based product	333–1821	Portugal	
	Maize grain	ND-700	Ethiopia	
	Sorghum grain	40–112	Ethiopia	
ZEA	Corn	59–505	Philippines	[63,64,65]
	Cereal and corn	5–930	Portugal	
	Sorghum grain	7.2–382	Ethiopia	
BEA	Rice	3800–26,300	Morocco	[46]
	Cereal	0.1–10,600	Morocco	
ENN A	Rice	8400–119,500	Morocco	[46]
ENN A1	Rice	56,200–448,700	Morocco	
ENN B	Rice	4400–26,200	Morocco	
ENN B1	Rice	3600–23,700	Morocco	
FB1	Maize	ND-1106	Zimbabwe	[3,63]
	Industrial processed food	43–836	Nigeria	
	Dried sweet potato chips	29.34–628.78	Tanzania	
	Corn	113–1162	Portugal	
	Corn products	183–2026	Portugal	
AOH	Tomato sauce	1.2–20.8	Europe	[46,64,66]
	Sunflower oil	0.7–2.9	Europe	
	Sorghum grain	75–1090	Ethiopia	
	Cereal	0.75–832	Germany	
	Fruit juices	15–100	Germany	
AME	Tomato sauce	<LOQ-4.7	Europe	[46,64,66]
	Sunflower oil	<LOQ-7.1	Europe	
	Sorghum grain	13–257	Ethiopia	
	Cereal	0.3–905	Germany	
	Fruit juices	0.13–4.9	Germany	
ALT	Tomato products	6.1–62	Belgium	[46]
	Fruit juices	1.18–18.4	Germany	
ATXs	Tomato sauce	0.5–3.7	Europe	[66]
	Sunflower oil	2–4.7	Europe	
TeA	Tomato sauce	<LOQ-691	Europe	[46,66]
	Sunflower oil	24–458	Europe	
	Fruit juices	1.1–250	Germany	
	Infant food	0.8–1200	Germany	
TEN	Tomato sauce	0.2–1.2	Europe	[46,66]
	Sunflower oil	<LOQ-21.8	Europe	
	Fruit juices	0.5–10.7	Germany	
AFB1	Polished rice	1–2546	Philippines	[64,65]
	Sorghum grain	<7.5–359	Ethiopia	
PAT	Apples	3.2–1500	Portugal	[63]
	Quince jam	9.7–28.7	Portugal	
OTA	Cereals	0.27–7.97	Portugal	[63,64,65]
	Coffee beans	8-36,561	Philippines	
	Sorghum grain	3.7–163	Ethiopia	

LOQ: Limit of quantitation; ND: Not detected.

**Table 3:** Advantages and disadvantages of in vivo and in vitro models in the evaluation of bioavailability.

Models	Advantages	Disadvantages
In vitro models		
Simulation of gastrointestinal transformation	Similar to the physiological processes in the human body Suitable for high-throughput format Ability of testing a specific mechanisms of action Focus on small number of components Validation with reference material	No hormonal and nervous control Lack of feedback mechanisms Absence of mucosal cell activity Deficiency of complexity of peristaltic movements, and involvement of the local immune system Homeostatic mechanisms are not present Difficult to achieve the anaerobic assay conditions
Caco-2 cells	Reproducibility of results Provides information about efficiency of digestion, absorption Ability of studying transport mechanisms Phenotypically similar to absorptive epithelial cells Suitable for high-throughput format	Human colonic adenocarcinoma origin Higher TEER value than human intestine Lack of mucin, microflora, biofilms, and epithelial cell types Variation of efflux transporters expression levels Incapability of simulating the changes of pH
In vivo models	In vivo condition Well-known biology Selection of specific subjects Better-understanding kinetic of mycotoxins	High-throughput limitation Extremely complex functional systems Influence of different factors-phenotypic variation Lack of certified reference standards Ethical issues and high cost Time consuming and labor intensive

TEER: Transepithelial electrical resistance.

**Table 4:** Available human and animal cell lines used for in vitro biotransformation and bioavailability of drugs and xenobiotics.

Cell line	Origin	Transporters, Enzymes and Other Relevant Proteins	References
Caco-2	Human colon adenocarcinoma	CYP1A1, 1A2 GST, UGT, SULT, NAT P-gp, MRP-2, BCRP	[76,77,78]
HT-29	Human colon adenocarcinoma	CYP2C8, CYP2J2, CYP3A4 GST, UGT MRP1, MRP2, p-gp, BCRP	[79,80,81]
TC-7	Caco-2 subclones	Similar to Caco-2	[82]
T84	Human colonic carcinoma	P-gp, MRP2, MRP3	[83,84]
H4	Human small foetal intestine	CYP3A4	[85]
IPEC-J2	Neonatal pig small intestine	CYP1A1, 1A2, 3A29 P-gp, MRP1, BCRP	[86,87]

**Table 5:** CYP450 isoforms induced by mycotoxins and their phase I and II metabolites.

Mycotoxins	Induced CYP450	Phase I Biotransformation	Phase II Biotransformation	References
T-2 and HT-2	CYP3A46, 3A29 and 3A22 in pig CYP1A5, 3A37 in chicken CYP1A1 in human	NEO, 3′-OH-T-2, 3′-OH-HT-2, T-2 triol, T-2 tetraol, and some C12,13-deepoxy products	T-2 glucuronides HT-2 glucuronides	[34,93,94,95,96,97,98,99,100,101]
DON	CYP2B1 and 2B2	DOM-1	DON-3-gluccoside, DON-, DOM- and DON -3-Glucoside-sulfonates, DON-3-, DON-7-, DON-8- and DON-15- glucuronides	[34,102]
ZEA	CYP1A1, 1A2, 2B6, 2C9, 3A4 and 3A5 in human CYP2C7, 2E1, 3A1 and 3A2 in rat	α-ZEA and β-ZEA	ZEA, α-ZEA and β-ZEA-glucuronides ZEA-14-Glucoside, α-ZEA-14-Glucoside, β-ZEA-14-Glucoside, ZEA-14-Sulfate and ZEA-16-Glucoside	[34,103,104,105,106]
BEA	CYP3A4/5 and CYP2C19 in human CYP3A1/2 in rat	No metabolites detected	No metabolites detected	[107,108]
ENNs	CYP3A4, 2C9, 1A2 in human CYP3A and 1A in rat and dog	M1–M12 with rat, dog and human liver microsomes M1–M5, M9–M13 in chicken	No sulfated or glucuronidated of ENN B and B1 detected	[109,110,111,112,113,114,115]
FB1	CYP 1A1 and 4A1 in rat CYP1B1 in human	HFB1 and pHFB1	Unknown	[34,116]
AOH and AME	CYP1A1	OH-AOH and OH-AME	AOH-3-glucoside, AOH-9-glucoside and AME-3-glucoside	[117,118,119,120]
ATXs	CYP1A1	ATX I	No metabolites detected	[121]
ALT	Unknown	OH-ALT	ALT-glucuronide	[121]
TeA	Unknown	No metabolites detected	No metabolites detected	[121]
TEN	CYP3A4	Monooxidized, mono-methylated and di-methylated metabolites	Unknown	[121]
AFB1	CYP1A1, 1A2, 2B6, 2C9, 3A4 and 3A5 in human liver	AFBO, AFM1, AFL, AFQ1 and AFP1	AFB1-glutathiones, glucuronides and sulfates	[34,122,123]
OTA	CYP1A1, 1A2, 2B6, 2C9, 3A4 and 3A5 in human liver	Lactone-open OTA, OTα, OTB, 4-OH-OTA and 10-OH-OTA	OTA-glutathiones, OTA-hexose/pentose, OTA-sulfates	[34,124,125,126,127,128,129]
PAT	CYP1A1, 1A2, 2B6, 2C9, 3A4 and 3A5 in human hepatocytes	E-ascladiol, Z-ascladiol, hydroascladiol and deosypatulinic acid	PAT-glutathiones	[34,130]

T-2 toxin (T-2), HT-2 toxin (HT-2), neosolaniol (NEO), deoxynivalenol (DON), zearalenone (ZEA), beauvericin (BEA), enniatins (ENNs), fumonisin B1 (FB1), alternariol (AOH), alternariol monomethyl ether (AME), altenuene (ALT), altertoxins (ATXs), tenuazonic acid (TeA), Tentoxin (TEN), aflatoxin B1 (AFB1), mycophenolic acid (MPA), ochratoxin A (OTA), patulin (PAT), 3′-hydroxy-T-2 (3′-OH-T-2), 3′-hydroxy-HT-2 (3′-OH-HT-2), hydroxy-alternariol (OH-AOH), hydroxy-alternariol monomethyl ether (OH-AME), deepoxy-deoxynivalenol (DOM-1), α-zearalenone (α-ZEA), β-zearalenone (β-ZEA), hydroxy-altenuene (OH-ALT), AFB1–8,9-epoxide (AFBO), aflatoxin M1 (AFM1), aflatoxicol (AFL), aflatoxin Q1 (AFQ1), aflatoxin P1 (AFP1), ochratoxin α (OTα), ochratoxin B (OTB), 4-hydroxy-OTA (4-OH-OTA) and 10-hydroxy-OTA (10-OH-OTA), cytochrome P (CYP).

**Table 6:** Studies investigating bioavailability of mycotoxins by Caco-2 cells.

Mycotoxins	Concentration (µM)	Incubation Time (h)	Major Findings	References
AOH and AME	20	1–3	22.7–25.8% and 3–7.1% applied AOH and AME reached the basolateral compartment (including their metabolites).	[119]
ATXs	10	0.5	6% and 0.3% applied ATX I and ATX II found in basolateral compartment. ATX I were not metabolized. 13 and 4% metabolites of ATX II found in apical and basolateral compartments.	[201]
AFB1	1–25	24–48	CYP1A2 and 3A4 were the main CYP450 isoforms for AFB1 activation into the genotoxic metabolite aflatoxin-exo-7-8-epoxyde.	[9]
AFB1, FB1, OTA and T-2	100	24	AFB1, FB1, T2 and OTA disrupted the intestinal barrier permeability.	[198]
BEA	1.5–3	4	Bioavailability was from 50.1–54.3 for BEA	
DON	5-30	24	DON transcellular passage was either by passive/facilitated diffusion or by active transport. DON was a substrate for both P-gp and MRP2.	[202]
ENNs	1.5–3	4 48	Duodenal bioavailability: 57.7–76.8% for ENN A, 68.8–70.2% for ENN A1, 65.0–67.0% for ENN B, and 62.2–65.1% for ENN B1. Colonic bioavailability: 17.3–33.3% for ENN A, 40.8–50.0% for ENN A1, 47.7–55.0% for ENN B, and 52.4–57.4% for ENN B1	[67]
MPA	0–780	-	Decrease in the barrier function of Caco-2 cell monolayer.	[9]
NIV	5	6	Bioavailability: 32.6% NIV would not be metabolized in Caco-2 cells. NIV was a substrate for P-gp and MRP2.	[203]
OTA	1–100 5–45	1 3–24	OTA was a substrate for MRP2 and BCRP Metabolites were OTB, OTA methyl ester, OTA ethyl ester and the OTA glutathione conjugate.	[204] [205]
ZEA	25	4	ZEA was substrates for ABCC1, ABCC2 and metabolites into α- and β-zearalenol and glucuronides.	[206]

Alternariol (AOH), alternariol monomethyl ether (AME), altertoxin (ATXs), aflatoxin B1 (AFB1), fumonisin B1 (FB1), ochratoxin A (OTA), T-2 toxin (T-2), beauverincin (BEA), deoxynivalenol (DON), enniatins (ENNs), mycophenolic acid (MPA), nivalenol (NIV), zearalenone (ZEA), cytochrome P (CYP), P-glycoprotein (P-gp), multidrug resistance protein (MRP), breast cancer resistance protein (BCRP), ATP-Binding Cassette (ABC).

## Abbreviations

3′-OH-HT-2	3′-hydroxy-HT-2
3′-OH-T-2	3′-hydroxy-T-2
4-OH-OTA	4-hydroxy-ochratoxin A
10-OH-OTA	10-hydroxy-ochratoxin A
α-ZEA	α-zearalenone
β-ZEA	β-zearalenone
ABC	ATP–binding cassette
AFB1	aflatoxin B1
AFB2	aflatoxin B2
AFBO	Aflatoxin B1–8,9-epoxide
AFBO-GSH	Aflatoxin B1–8,9-epoxide-glutathiones
AFG1	aflatoxin G1
AFG2	aflatoxin G2
AFL	aflatoxicol
AFM1	aflatoxin M1
AFM2	aflatoxin M2
AFP1	aflatoxin P1
AFQ1	aflatoxin Q1
AhR	aryl the hydrocarbon receptor
AKR7	aldo-keto reductase subfamily 7
ALT	altenuene
AME	alternariol monomethyl ether
AOH	alternariol
AP	apical compartment
ATX I	altertoxin I
ATX II	altertoxin II
ATXs	altertoxins
BCRP	breast cancer resistance protein
BEA	beauvericin
BL	basolateral compartment
Caco-2	caucasian colon adenocarcinoma
Caco-2/TC7	TC7 clone was isolated from a late passage of the parental Caco-2 line
CAR	constitutive androstane receptor
CYP	cytochrome P
D3G	deoxynivalenol-3-glucoside
DNA	Deoxyribonucleic acid
DOM-1	deepoxy-deoxynivalenol
DON	deoxynivalenol
ENN A	enniatin A
ENN A1	enniatin A1
ENN B	enniatin B
ENN B1	enniatin B1
ENNs	enniatins
ERK	extracellular signal regulated protein kinase
FB1	fumonisin B1
FBs	fumonisins
GI	gastrointestinal
GSH	glutathione
GST	glutathione S-transferase
HCT-16	human colon carcinoma
HFB1	aminopentol
HT-2	HT-2 toxin
HT-29	human colorectal adenocarcinoma
IL-8	Interleukin-8
JNK	c-Jun-N-terminal kinase
LD_50_	median lethal dose
LOQ	limit of quantitation
MAPK	mitogen-activated protein kinase-dependent
mEH	microsomal epoxide hydrolase
MPA	mycophenolic acid
MRP	multidrug resistance protein
NAT	N-acetyltransferaseND (not detected)
NEO	neosolaniol
NF-κB	nuclear factor kappa–light–chain–enhancer of activated B cells
NIV	nivalenol
OH-ALT	hydroxy-altenuene
OH-AME	hydroxy-alternariol monomethyl ether
OH-AOH	hydroxyl-alternariol
OTα	ochratoxin α
OTA	ochratoxin A
OTB	ochratoxin B
P_app_	apparent permeability
P-gp	P-glycoprotein
PAT	patulin
pHFB1	partially hydrolyzed fumonisin B1
PXR	pregnane X receptor
RNA	Ribonucleic acid
ROS	reactive oxygen species
SULT	sulfotransferase
SW480	human colon adenocarcinoma
T-2	T-2 toxin
TeA	tenuazonic acid
TEER	transepithelial electrical resistance
TEN	tentoxin
UGT:	uridine 5′-diphospho-glucuronosyltransferase
ZEA	zearalenone
ZEA14Glc	zearalenone-14-glucoside
ZEA16Glc	zearalenone-16-glucoside
